# Concurrent Changes in 24-Hour Movement Behaviors and Cognitive Function during Retirement Transition: Longitudinal Compositional Data Analysis

**DOI:** 10.1249/MSS.0000000000003801

**Published:** 2025-07-04

**Authors:** LOTTA PALMBERG, KRISTIN SUORSA, TUIJA LESKINEN, JESSE PASANEN, SUVI ROVIO, SARI STENHOLM

**Affiliations:** 1 Department of Public Health, University of Turku and Turku University Hospital, Turku, FINLAND; 2 Centre for Population Health Research, University of Turku and Turku University Hospital, Turku, FINLAND; 3 Gerontology Research Center and Faculty of Sport and Health Sciences, University of Jyväskylä, Jyväskylä, FINLAND; 4 Research Center of Applied and Preventive Cardiovascular Medicine, University of Turku, Turku, FINLAND; 5 Research Services, Turku University Hospital and University of Turku, FINLAND

**Keywords:** COGNITIVE FUNCTION, RETIREMENT, ACCELEROMETRY, SLEEP, SEDENTARY TIME, PHYSICAL ACTIVITY, COMPOSITIONAL DATA ANALYSIS, AGING

## Abstract

**Background:**

Transitioning to retirement may change physical activity, sedentary behavior, and sleep, i.e., 24-h movement behaviors, but it is unknown whether these changes are linked to cognitive function. This study investigates the longitudinal associations between changes in 24-h movement behaviors and cognitive function during the retirement transition.

**Methods:**

Study population consisted of public sector workers (*n* = 146; mean age, 63.3 yr; SD, 1.0) from the Finnish Retirement and Aging study. A thigh-worn Axivity accelerometer was used to estimate daily time in sleep, sedentary behavior (SED), light physical activity (LPA), and moderate-to-vigorous physical activity (MVPA) before and after retirement (1 yr in between). Similarly, computerized Cambridge Neuropsychological Test Automated Battery was conducted repeatedly to evaluate six cognitive domains: learning and memory, working memory, sustained attention and information processing, executive function and cognitive flexibility, and reaction time. Associations between changes in 24-h movement behaviors and cognitive function were analyzed using compositional linear regression and isotemporal substitution analyses.

**Results:**

An increase in active (LPA and MVPA) relative to passive behaviors (sleep and SED) and SED relative to sleep were associated with improvement in reaction time (β_ilr_ = 0.21, *P* = 0.04, β_ilr_ = 0.55, *P* = 0.02). Especially reallocating time from sleep to other behaviors showed positive associations. For instance, reallocating 30 min from sleep to other behaviors was associated with 0.05 standardized unit improvement in reaction time. No associations between changes in movement behaviors and changes in any other cognitive domain were observed.

**Conclusions:**

Reallocating time from sleep to other behaviors during retirement transition was associated with improvement in reaction time. Further studies are needed to examine long-term consequences of changes in 24-h movement behaviors for cognitive function.

Cognitive function declines and the prevalence of cognitive impairment increases with advancing age. Maintaining sufficient cognitive functioning is essential for independent living and good quality of life (1,2). While old age is a primary risk factor for cognitive impairment, low educational level, unhealthy lifestyle, lack of cognitively stimulating activities, and chronic conditions are linked to increased risk of cognitive impairment and dementia (3). Moreover, different life events in late adulthood, such as widowhood or retirement which are accompanied with changes in above mentioned risk factors, are suggested to associate with changes in cognitive functioning and risk of dementia (4–8).

Retirement may accelerate decline in cognitive functioning in some domains such as episodic and verbal memory over several years after retirement (5–9), but findings are inconclusive and may be moderated by factors, such as job strain and motivation, to maintain engagement to cognitively challenging activities after retirement (5,7). In contrast, our recent findings from the Finnish Retirement and Aging study suggest that retirement transition is associated with short-term improvement in several domains of cognitive function including learning and memory, working memory, sustained attention, and information processing (10).

Physical activity, sedentary behavior, and sleep are also shown to change during the retirement transition (11). All these movement behavior components have been linked with cognitive function. Especially for physical activity, both longitudinal and experimental studies consistently support associations with improved cognitive function in older cohorts (12–15). On the other hand, both short and long sleep duration have been associated with poorer cognitive function (16–18), while findings on objectively measured sleep duration and changes in cognitive function are conflicting (18–21). Prior evidence on the associations between sedentary behavior and cognitive function is inconclusive and potentially differ by cognitively active versus passive sedentary behavior (19).

Importantly, the majority of earlier studies about the association of physical activity, sedentary behavior, and sleep with cognitive function have studied each movement behavior separately. They have thus ignored that these behaviors happen within the constraints of the 24-h day, and changes in one behavior component will necessarily lead to changes in at least one other behavior. A few earlier studies examining the association of movement behaviors with cognitive function in midlife and old age have taken into account the codependency between movement behaviors. Moderate-to-vigorous physical activity (MVPA) relative to other movement behaviors was found to associate with better global cognition, language, memory, and executive function among middle-aged and older adults (22–24). In addition, Mellow and colleagues reported an association between higher MVPA and better long-term memory only among those older adults with smaller than average frontal lobe volume (25).

The findings on the relative association of sedentary behavior and sleep with cognitive function are more inconclusive. Mitchell and colleagues, and Whitaker and colleagues, found a positive association between higher relative sedentary time and better cognitive function among middle-aged adults (23,24), while Feter and colleagues reported an association between lower sedentary time and better performance on several domains of cognitive function such as memory, language and executive functions among those middle-aged and older adults with short sleep duration (20). However, lower sedentary time and higher sleep duration were observed to associate with lower cognitive function among those with sufficient sleep duration (20). Furthermore, a positive association between sleep duration and executive function and negative association between sedentary time and executive function were found among older adults with smaller than average total gray matter volume (25). These earlier studies, however, are limited by either a cross-sectional study design or not considering sleep in the movement behavior composition, and most by focusing only on certain cognitive domains.

Since 24-h movement behaviors and cognitive function are both shown to change during retirement transition, it is of interest to examine whether changes in daily movement behavior composition during retirement may underlie our prior observation on the positive change in cognitive function immediately after retirement transition (22). Retirement transition can be utilized as a natural experiment setting enabling to study how changes in one health indicator associates with changes in another indicator.

Hence, the aim of this longitudinal study was to examine whether changes in 24-h movement behaviors relate to changes in several domains of cognitive function during a 1-yr retirement transition using compositional data analysis. The secondary aim was to examine associations separately for changes in 24-h movement behaviors from workdays before retirement to all days after retirement and from non-work days before retirement to all days after retirement, because the proportions of 24-h movement behaviors have been observed to be markedly different between workdays and days off (23,24,26).

## METHODS

### Study design and participants

The study population consisted of participants from the Finnish Retirement and Aging Study (FIREA), an ongoing longitudinal cohort study of older adults in Finland established in 2013. Details of the design and implementation of the FIREA study have been reported elsewhere (27). Shortly, participants were first contacted 18 months before their estimated retirement date by sending them a questionnaire. After responding to the questionnaire, Finnish-speaking participants with estimated retirement date between 2017 and 2019, who lived in Southwest Finland and were still working, were invited to participate in the clinical substudy (*n* = 773). Of them, 290 agreed to participate. Thereafter, study participants were followed up with annual measurements, including questionnaires, clinical, and accelerometer measurements. To determine the timing of retirement, the actual retirement day was inquired during each phase of the data collection, and this information was used to determine pre-retirement and post-retirement measurements.

Of the clinical substudy participants, 240 took part in accelerometer and cognitive measurements before and after the transition to full-time statutory retirement, with on average 1 yr in between the measurements. We excluded participants who had less than three valid accelerometer measurement days before and/or after retirement and less than one valid workday and one valid non-work day before retirement (n = 85), as well as missing information on cognitive function (n = 9–10). Thus, the analytical sample was 146 participants for all other cognitive domains except for reaction time for which valid data was available from 145 participants (Supplemental Fig. 1, Supplemental Digital Content http://links.lww.com/MSS/D270).

### Assessment of 24-h movement behaviors

To estimate 24-h movement behaviors; sleep, sedentary time (SED), light physical activity (LPA) and MVPA, participants wore a triaxial accelerometer Axivity AX3 (Axivity Ltd Newcastle, UK) on the thigh. Detailed description of the measurement protocol is reported elsewhere (28). Briefly, the accelerometer was fastened to the skin of the front of the right thigh, midway between iliac crest and the upper line of patella with adhesive waterproof film dressing during the clinical visit. Participants were instructed to wear the accelerometer 24 h·d^−1^ without breaks and fill out a daily diary. Before retirement, participants were asked to wear the accelerometer at least 4 days and nights, including at least two workdays and two non-work days, and after retirement at least four days and nights. During the measurement period, participants were instructed to perform a reference measurement in a standing upright position for 15 s each day and record the time of the reference measurement, and also the waking time, time of going to bed, and start and end of the work interval on workdays into the diary.

Data from the accelerometers were downloaded through Open Movement software (version 1.0.0.37; Open Movement, Newcastle University, UK). The raw data were processed to determine 24-h movement behaviors using a customized MATLAB program, ActiPASS (version 0.80) (29), an automatized version of Acti4, which determines the type and duration of different activities and body postures based on threshold values of standard deviation of acceleration and the derived inclination with a high sensitivity and specificity (30,31). Measurement period was restricted to days between the first and last date and time recorded in the daily diary. Non–wear time was detected using algorithm in the ActiPASS (≥60 min periods without movement) (31). The measurement day was determined from midnight to midnight and a valid measurement day was defined as a day with at least 10 h of wear time during waking hours.

ActiPASS was used to determine time spent sitting, lying (32), standing still, moving, walking slow (<100 steps per min) and fast (≥100 steps/minute), running, cycling, stair climbing and in other physical activity (30,31). Time spent in sitting and lying down were merged into SED, standing still, moving, and walking slow were merged into LPA and the rest of the activity types were merged into MVPA (28). Information on sleep was obtained from the daily diary and sleep time was defined as a period between going to and out of the bed. Weekly means of wake-time behaviors were calculated from all valid days and weekly means of sleep from all nights with daily log-determined waking and bedtimes.

### Assessment of cognitive function

Cognitive function was assessed with identical protocol before and after retirement with five tests from the Cambridge Neuropsychological Test Automated Battery (CANTAB®). The cognitive test was conducted using a touch-screen computer and administered by a trained research nurse during the clinical study visit. CANTAB® is a standardized computerized methods for testing cognitive function that includes multiple tests that assess different domains of cognitive function. The test battery included tests in the following domains: (1) *reaction time* was assessed with Reaction Time test including measurement of reaction time and movement time and response accuracy, 2) *executive function and cognitive flexibility* was assessed with Attention Switching Task including aspects of set-shifting and inhibitory control, 3) *working memory* with the Spatial Working Memory test measuring short-term and spatial working memory and problem solving, 4) *learning and memory* with the Paired Associates Learning test measuring visual and episodic memory and visuospatial associative learning, and 5) *sustained attention and information processing* with the Rapid Visual Information test assessing visual processing, recognition and sustained attention.

Due to a high number of outcome variables from each test, we used a standardized classification-based summary score for each test to obtain scores that would explain most of the variation within each domain. For the study baseline data, all individual outcome variables were standardized into a scale with a mean of 0 and standard deviation of 1, and if needed transformed so that higher values correspond to better cognitive function. Summary scores for each test were then calculated by summing up the values of their respective standardized variables and dividing the sum by the number of variables in the test. For the follow-up measurements, the standardization was instead conducted with respect to the baseline distribution (mean and standard deviation), and the summary scores were calculated using these values. The change in cognitive function during retirement transition was calculated as the difference between each respective test score after and before retirement. Detailed description of the outcome variables used to create summary scores for each domain are provided in Supplemental Table 1 (Supplemental Digital Content http://links.lww.com/MSS/D270).

### Assessment of preretirement characteristics

Sex, date of birth, and pre-retirement occupational title were obtained from the Keva Public Sector Pensions register. Participants were divided into two occupational status groups according to the occupational titles of the last known occupation preceding retirement by using the International Standard Classification of Occupations (ISCO): manual workers (e.g., cleaners, maintenance workers to ISCO classes 5–9) and non-manual workers (e.g., teachers, physicians, registered nurses, technicians to ISCO classes 1–4).

Information on other characteristics were obtained from the questionnaire preceding the transition to retirement. Marital status was categorized as single (unmarried, divorced and widows) and married/cohabiting. Job strain was measured with Job Content Questionnaire (33) and participants were dichotomized into with or without job strain as previously (23). Depression which was identified using the Beck Depression Inventory (34) and used as a continuous and categorized measure (no depression: ≤9 points, depression: >9 points (mild/moderate, there were no participants with severe depression). Heavy alcohol consumption was indicated by self-reported average consumption of more than 288 g·wk^−1^ of pure alcohol for men and 192 g·wk^−1^ for women (Anderson & Colom, 2015). Self-reported doctor-diagnosed cardiovascular diseases (angina pectoris, myocardial infarction, cerebrovascular disease, claudication) were categorized as no/yes (one or more).

To examine selection to the current study, the pre-retirement participant characteristics were compared between the current study population (*n* = 146) and larger FIREA survey cohort (*n* = 3698). In these analyses, we used health-related characteristics, which were available from the FIREA survey cohort participants. In addition to previously mentioned job strain and alcohol consumption, doctor-diagnosed depression, sleep duration (hours per night), sitting time (sum of daily hours spent sitting at work, watching television, using computer at home, sitting in a vehicle and other sitting) (35) and leisure-time and commuting physical activity as metabolic equivalents (MET) hours per week (36) were included.

### Statistical analyses

Descriptive information on participant characteristics is presented using means and standard deviations for continuous variables and frequencies and percentages for categorical variables. Changes in participant characteristics were examined using McNemar test for categorical variables and linear mixed models for continuous variables. Comparison between participants included in the present analyses and survey study population who also had pre-retirement and post-retirement information available were analyzed using χ^2^ test for categorical variables and ANOVA for continuous variables.

The 24-h movement behavior data was treated as compositional data, normalized to 24 h·d^−1^. The statistical analyses were conducted using R software (version 4.3.1, R Foundation for Statistical Computing, Vienna, Austria). The dataset did not include zero values for 24-h movement behaviors, and thus no imputation was needed. An isometric log ratio (ilr) transformation was used to map the compositional data into real-valued coordinates, which reduces the dimensionality of the data and allows standard statistical methods to be used (37). The specific type of ilr coordinates used in this study were *balance coordinates* (38). The balance coordinates were formed using sequential binary partitioning: first, active behaviors, that is, combination of LPA and MVPA, were selected as positive (placed in the numerator of the balance coordinate 1), and the passive behaviors, that is, combination of sleep and SED, were selected as negative (placed in the denominator of the balance coordinate 1). The positive values of this coordinate indicates that the relative contribution of active behaviors exceeds that of passive behaviors, and vice versa. For the second balance coordinate, LPA was selected as positive and MVPA as negative. Finally, for the third balance coordinate, SED was selected as positive and sleep as negative. Detailed description of the ilr coordinate setup is provided in Supplemental Material 1 (Supplemental Digital Content http://links.lww.com/MSS/D270). Change in 24-h movement behavior composition was calculated by subtracting each balance coordinate after retirement from balance coordinates before retirement.

We examined associations between change in 24-h movement behaviors and change in five cognitive function domains during the transition to retirement using compositional linear regression models (Supplemental Material 1, Supplemental Digital Content http://links.lww.com/MSS/D270). We made *a priori* assumption that changes in 24-h movement behaviors, especially in physical activity, would drive changes in cognitive function based on previous literature (12,13,15). Thus, changes in cognitive domains were used as the dependent variables and change in 24-h movement behaviors (expressed as balance coordinates) as the independent variables. In Model 1, covariates included 24-h movement behavior composition (expressed as balance coordinates) and cognitive function before retirement, as well as age, sex and occupational status. Model 2 was additionally adjusted for pre-retirement marital status, job strain and depression (as continuous variable). In addition, to account for possible differences between workdays and non-work days, associations were examined separately for change in 24-h movement behaviors from workdays before retirement to all days after retirement and from non-work days before retirement to all days after retirement. The associations were presented as beta coefficients and their 95% confidence intervals (95% CI). The beta coefficients indicate the change in dependent variable for each one-unit balance coordinate increase, thus pointing out to presence of association but effect sizes cannot be drawn directly from the beta coefficients.

To aid interpretation of the findings and to calculate effect sizes, the effect of observed reallocations between 24-h movement behaviors on cognitive function were illustrated using the compositional isotemporal substitution model (39). First, systematic reallocations between movement behaviors were calculated based on the mean composition before retirement (i.e., 8.2 h sleep, 9.8 h SED, 4.7 h LPA and 76 min MVPA). The size of the reallocations was chosen based on the observed actual range of change in MVPA, between −60 and 60 min. The 60-min size of reallocations were used also for reallocations between sleep, SED and LPA to aid comparison of effect sizes between one-to-one reallocations between behaviors (e.g., to compare effect sizes between reallocating time from SED to MVPA vs SED to LPA). These reallocated compositions were then transformed to balance coordinates using the method explained above. Second, the regression-based coefficients from Model 1 (age, sex and occupation as covariates) were applied on the calculated change in balance coordinates to predict changes in cognitive function corresponding to changes in composition of 24-h movement behaviors during transition from work to retirement. Following the procedure applied previously (22), the observed change in cognitive function associated with no change in 24-h movement behavior composition was subtracted from the predicted changes in cognitive function. We did this to isolate the effects of one-to-one reallocations between 24-h movement behaviors only. The results are shown as estimated change in each cognitive domain and their 95% CI. When the CI did not include 0, the change was considered statistically significant.

Finally, given that associations between changes in 24-h movement behaviors and cognitive function may differ depending on whether sleep is increased/decreased from insufficient or sufficient level (18,20), we conducted a sensitivity analysis by excluding those reporting more than 9 h of sleep per night before retirement (*n* = 16, 11%) from the linear regression models.

## RESULTS

Descriptive characteristics of the study population before and after retirement are presented in Table 1. Of the participants, 82% were female, 68% were non-manual workers and their mean age before retirement was 63.3 yr (SD, 1.0). Before retirement, 13% reported job strain, 18% had depression (mild/moderate), 4% had cardiovascular disease, and 4% reported heavy alcohol consumption. The prevalence of depression was lower after retirement when compared with before retirement (10%, *P* = 0.002), but the prevalence of job strain, cardiovascular disease and heavy alcohol consumption remained similar. Domains of cognitive function improved from before retirement to after retirement, except for reaction time that showed no significant change.

**TABLE 1:** Characteristics of the study population (n = 146) before and after retirement.

Characteristics	Before Retirement	After Retirement	Change, *P*
Age, mean (SD)	63.3 (1.0)	64.4 (1.0)	—
Female, *n* (%)	120 (82)		—
Marital status, *n* (%)			1.00
Single	35 (24)	33 (24)	
Married/cohabitation	107 (75)	105 (76)	
Occupational group, *n* (%)			—
Manual	46 (32)		
Non-manual	100 (68)		
Job strain, *n* (%)	18 (13)		—
Depression, *n* (%)	27 (18)	14 (10)	0.002
Heavy alcohol consumption, *n* (%)	6 (4)	5 (4)	0.69
Cardiovascular disease*^a^*, *n* (%)	6 (4)	8 (6)	1.00
Cognitive outcomes*^b^*			
Reaction time, mean (SD)	−0.05 (0.55)	0.001 (0.35)	0.32
Executive function and cognitive flexibility, mean (SD)	0.11 (0.63)	0.22 (0.50)	0.003
Working memory, mean (SD)	0.05 (0.57)	0.16 (0.48)	0.02
Learning and memory, mean (SD)	0.06 (0.66)	0.25 (0.65)	<0.0001
Sustained attention and information processing, mean (SD)	0.08 (0.69)	0.21 (0.63)	0.001
Accelerometer measurements			
No. valid measurement days, mean (SD)	4.8 (0.99)	4.5 (0.69)	0.004
No. valid workdays, mean (SD)	2.5 (0.86)	-	—
No. non-work days, mean (SD)	2.3 (0.92)	-	—
No. daily log-determined nights, mean (SD)	3.3 (0.92)	3.1 (0.68)	0.19
Wear time during waking hours, h, mean (IQR)	15.6 (15.1–16.1)	15.2 (14.7–15.8)	<0.0001
Compositional mean of sleep, SED, LPA and MVPA, min	493, 588, 283, 76	518, 579, 269, 74	—

*^a^*Including angina pectoris, myocardial infarction, cerebrovascular disease, claudication.

*^b^*Standardized units.

SD, standard deviation; IQR, interquartile range.

Among the current study population job strain tended to be lower (14% vs 20%) and self-reported non-occupational physical activity higher (26.7 vs 23.4 MET h·wk^−1^) compared with survey study population (Supplemental Table 2, Supplemental Digital Content http://links.lww.com/MSS/D270), but no other differences in baseline characteristics were observed.

Table 2 presents the longitudinal associations between the 1-yr changes in 24-h movement behaviors (expressed as balance coordinates; active vs passive, LPA vs MVPA, SED vs sleep) and the changes in domains of cognitive function from work to retirement. In these analyses, the only cognitive domain that showed significant associations was reaction time. An increase in active behaviors (LPA and MVPA) in relation to passive behaviors (sleep and SED) was associated with an improvement in reaction time (β_ilr_ = 0.21, *P* = 0.04) (i.e., increase in reaction time test score). Also, an increase in SED in relation sleep was associated with an improvement in reaction time (β_ilr_ = 0.55, *P* = 0.02). After additional adjustments for pre-retirement marital status, job strain, and depression, associations for change in active behaviors in relation to passive behaviors and reaction time attenuated slightly (β_ilr_ = 0.19, *P* = 0.06) while the association for SED in relation sleep remained similar (β_ilr_ = 0.50, *P* = 0.04, Table 2).

**TABLE 2:** Associations between changes in 24-h movement behaviors (expressed as changes in balance coordinates from all days before retirement to all days after retirement) and changes in domains of cognitive function.

	Reaction Time		Executive Function and Cognitive Flexibility		Working Memory		Learning and Memory		Sustained Attention and Information Processing	
	*n* = 145		*n* = 146		*n* = 146		*n* = 146		*n* = 146	
	β_ilr_ (95% CI)	*p*	β_ilr_ (95% CI)	p	β_ilr_ (95% CI)	p	β_ilr_ (95% CI)	p	β_ilr_ (95% CI)	*p*
Model 1*^a^*										
Active vs passive	**0.21 (0.005–0.41)**	**0.04**	0.07 (−0.15 to 0.29)	0.53	−0.15 (−0.41 to 0.11)	0.25	−0.10 (−0.38 to 0.17)	0.47	−0.03 (−0.29 to 0.23)	0.81
LPA vs MVPA	0.07 (−0.20 to 0.30)	0.57	−0.05 (−0.33 to 0.23)	0.73	−0.21 (−0.53 to 0.12)	0.22	0.06 (−0.28 to 0.41)	0.72	0.03 (−0.30 to 0.36)	0.86
SED vs sleep	**0.55 (0.01–0.95)**	**0.02**	−0.29 (−0.82 to 0.21)	0.27	−0.07 (−0.68 to 0.54)	0.83	−0.08 (−0.73 to 0.56)	0.80	0.03 (−0.57 to 0.64)	0.92
Model 2*^b^*										
Active vs passive	0.19 (−0.01 to 0.40)	0.06	0.05 (−0.18 to 0.27)	0.68	−0.19 (−0.46 to 0.07)	0.16	−0.17 (−0.45 to 0.11)	0.24	−0.04 (−0.30 to 0.22)	0.76
LPA vs MVPA	0.08 (−0.20 to 0.36)	0.56	−0.03 (−0.34 to 0.28)	0.86	−0.24 (−0.61 to 0.13)	0.20	−0.06 (−0.44 to 0.32)	0.76	0.09 (−0.27 to 0.45)	0.62
SED vs sleep	**0.50 (0.02–0.98)**	**0.04**	−0.32 (−0.85 to 0.22)	0.24	−0.18 (−0.81 to 0.45)	0.58	−0.08 (−0.74 to 0.57)	0.80	−0.04 (−0.66 to 0.59)	0.91

*^a^*Adjusted for pre-retirement domains of cognitive function, pre-retirement 24-h movement behavior composition, age, sex and occupation.

*^b^*Adjusted for pre-retirement domains of cognitive function, pre-retirement 24-h movement behavior composition, age, sex, occupation, pre-retirement marital status, pre-retirement job strain and pre-retirement depression (as continuous variable).

β_ilr_ coefficients indicate the change in outcome for each 1-unit balance coordinate increase, thus pointing out to presence of association but effect sizes cannot be drawn directly from coefficients.

**FIGURE 1:**
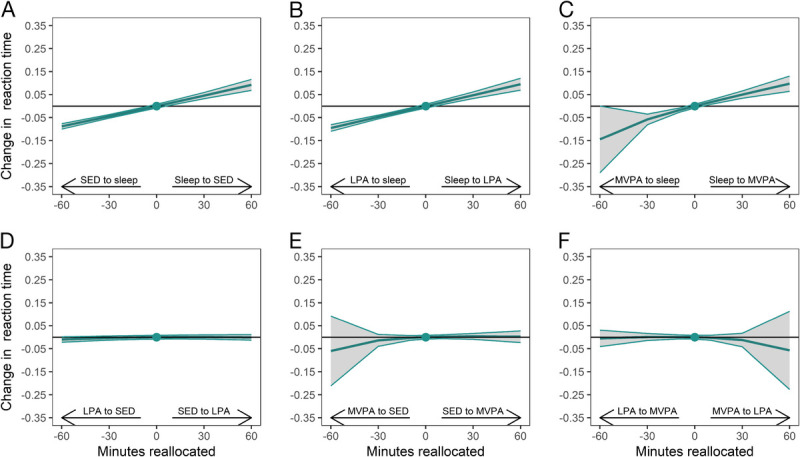
One-to-one reallocations between 24-h movement behaviors and changes in reaction time over 1 yr from work to retirement. The dot at 0 indicates the mean pre-retirement composition of 8.2 h sleep, 9.8 h SED, 4.7 h LPA and 76 min MVPA and reaction time of −0.05 (standardized value).

The observed association between 24-h movement behaviors and reaction time is illustrated in Figure 1 showing estimated effect of one-to-one reallocations between 24-h movement behaviors on reaction time. Reallocation of time from sleep to other behaviors was associated with an improvement in reaction time test score. For instance, reallocation of 30 min from sleep to SED, LPA or MVPA was associated with on average 0.05 standardized unit improvement in reaction time (Fig. 1 a, b, c). In contrast, reallocation of 30 min from SED, LPA or MVPA to sleep was associated with 0.04 to 0.06 standardized unit decline in reaction time, implying a linear association between changes in sleep in relation to the remaining behaviors and changes in reaction time. Reallocations between LPA and SED, MVPA and SED, LPA and MVPA were not associated with significant changes in reaction time (Fig. 1 d, e, f).

**FIGURE 2:**
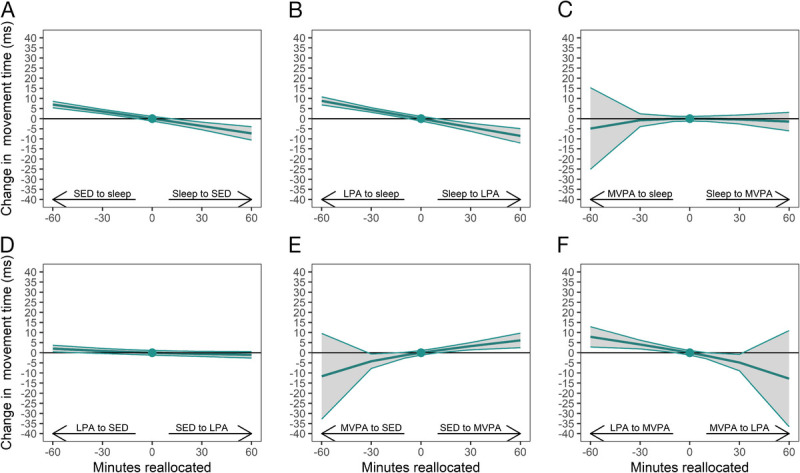
One-to-one reallocations between 24-h movement behaviors and changes in movement time (component of the reaction time test) over 1 yr from work to retirement. The dot at 0 indicates the mean pre-retirement composition of 8.2 h sleep, 9.8 h SED, 4.7 h LPA and 76 min MVPA and movement time of 309 milliseconds (ms).

**FIGURE 3:**
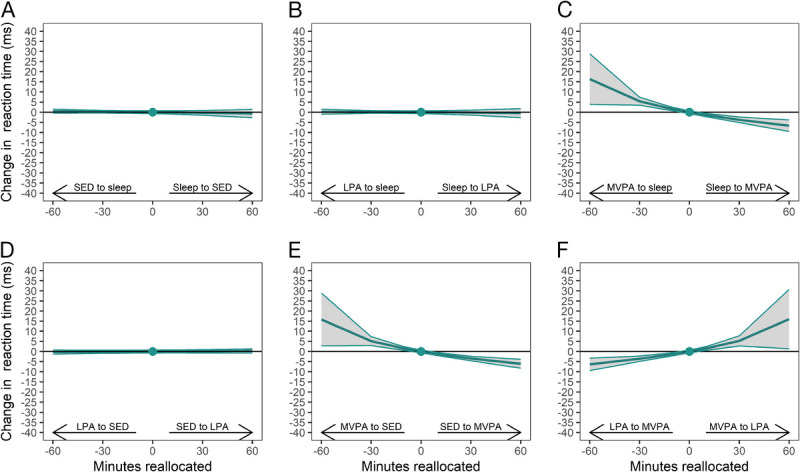
One-to-one reallocations between 24-h movement behaviors and changes in reaction time (component of the reaction time test) over 1 yr from work to retirement. The dot at 0 indicates the mean pre-retirement composition of 8.2 h sleep, 9.8 h SED, 4.7 h LPA and 76 min MVPA and reaction time of 307 milliseconds (ms).

To distinguish the estimated effect of one-to-one reallocations on the components of the reaction time test, i.e., movement time and reaction time, we conducted *post hoc* analyses, and their results are illustrated in Figure 2 and Figure 3. For movement time, especially reallocation of time from sleep to SED or LPA was associated with an improved performance (i.e., decrease in movement time) (Fig. 2, a, b). For instance, reallocation of 30 min from sleep to SED or LPA was associated with 3.6–4.3 ms decrease in movement time. For reaction time, only reallocation of time to MVPA from the other behaviors was associated with an improved performance (i.e., decrease in reaction time) (Fig. 3 c, e, f). For instance, reallocation of 30 min from other behaviors to MVPA was associated with 3.4–3.7 ms decrease in reaction time.

To account for possible differences between workdays and non-work days, the associations between changes in 24-h movement behaviors from workdays before retirement to after retirement and from non-work days before retirement to after retirement were also examined. However, there were no marked differences between associations for changes from workdays and non-work days (Supplemental Table 3, Supplemental Digital Content http://links.lww.com/MSS/D270).

To consider the possibility of the baseline sleep duration affecting the findings, a sensitivity analysis excluding the long sleepers (>9 h, *n* = 16) was carried out. The associations attenuated slightly (e.g., Model 2: active vs passive behaviors β_ilr_ decreased from 0.19 to 0.18, SED vs sleep β_ilr_ decreased from 0.55 to 0.41), but the direction of the associations did not change after excluding the long sleepers (Supplemental Table 4, Supplemental Digital Content http://links.lww.com/MSS/D270).

## DISCUSSION

The main finding of the present study was that increasing active behaviors (MVPA and LPA) relative to passive behaviors (SED and sleep) was associated with concurrent improvement in reaction time during the retirement transition. Further one-to-one time reallocations revealed that the association with reaction time was mainly driven by changes in sleep duration, and reallocating time from sleep into other behaviors (MVPA, LPA, and sedentary behavior) was associated with improvement in reaction time. These findings were similar for changes from both workdays and non-work days before retirement. The finding was exclusive for reaction time as we found no association between changes in movement behaviors and changes in any other domain of cognitive function.

To our knowledge, this is the first study to examine the longitudinal associations between the composition of 24-h movement behaviors and cognitive function. We utilized unique study setting in which participants have been followed with repeated measurements before and after retirement. Since transitioning from work to retirement has shown to modify 24-h movement behaviors as well as cognitive function (11,22), studying concurrent changes in 24-h movement behavior composition and cognitive function could shed light on the intricate association between different movement behaviors and specific cognitive domains.

We found that the association of changes in 24-h time-use composition and change in reaction time during retirement transition was mainly driven by changes in sleep duration. The mean composition before retirement included 8 h 13 min of sleep, which is likely sufficient for most participants. Increasing sleep from the sufficient level unlikely brings any additional benefits, on the contrary, it may take time away from cognitive stimulating activities or implicate poor sleep quality. Our findings are consistent with previous findings showing that hypothetical increase of sleep from sufficient level (7 h or more) is associated with poorer cognitive performance (20) and that long sleep duration (≥9 h) is associated with poorer reaction time (40,41). One potential reason underlying the association between longer sleep duration and cognitive function is that long sleep duration may reflect sleep problems or poor sleep quality (42), i.e., sleeping longer does not always increase restorative sleep. Since the focus of the current study was on the time spent in different movement behaviors, including sleep, we cannot be certain whether increase in sleep duration during retirement transition was also accompanied with concurrent changes in sleep quality. Moreover, given that the number of short and long sleepers were low in our study, future studies are needed to elucidate the role of sleep duration and quality on the association between sleep and cognitive function.

Reallocation from sleep into sedentary behavior was also associated with improvement in reaction time. This finding is consistent with earlier findings by Mitchell and colleagues who found that higher sedentary behavior was associated with better cognitive function among middle-aged adults (23). Furthermore, Whitaker and colleagues found that among middle-aged men replacing sedentary behavior with LPA was associated with unfavorable changes in cognitive function, but the authors did not include sleep into the models (24). The association of sedentary behavior and cognitive function may also depend on the type and context of sedentary behavior. Sedentary time may include several cognitively stimulating activities such as doing Sudoku and crosswords, which may support or even improve cognition, whereas excessive amounts of cognitively passive sedentary time, such as watching TV, could have detrimental effect on cognition (19). This finding warrants further research, as we used accelerometer-based information to estimate sedentary behavior, and therefore did not have information on the type and context for sedentary behaviors.

Further exploration into components of reaction time showed that changes in sleep duration relative to other behaviors (LPA and SED) drove changes in movement time, but not in reaction time. This finding may partly be explained by movement time potentially being more sensitive to changes in sleep duration than reaction time (43). Movement time has been traditionally thought to describe solely motor execution speed but may also reflect information processing (44). In contrast, we found that reallocation of time into MVPA was associated with improvement in reaction time i.e., time from stimulus to the initiation of movement, but not in movement time. This is consistent with earlier studies that have found associations between higher physical activity and better reaction time (45,46). For instance, O’Brien and colleagues found that older adults who met the physical activity guidelines had faster reaction time compared with those who did not (46). Potential biological mechanisms for the positive association between MVPA and cognitive processes may act through improved blood circulation of the brain and neurogenesis (47). Furthermore, while increasing physical activity alone can improve reaction time (45), a combination of physical and cognitive exercises may be even more beneficial for older adults' reaction time (45,48). While we were not able to assess different activity types, many types of MVPA such as dancing and ball games can be considered simultaneously physically, cognitively and socially stimulating. Finally, higher muscle strength and cardiovascular fitness level has also been associated with faster reaction times (49,50), which may also explain the finding.

Of the several domains of cognitive functions examined, we observed concurrent changes only with reaction time. Earlier cross-sectional studies have reported associations between 24-h movement behaviors with several domains of cognitive function such as language, memory, and executive functions (22,23). One potential explanation why these associations were not observed in our study could be short follow-up period of only 1 yr directly targeting retirement transition period, which may not have been sufficiently long in detecting changes in cognitive function attributable to the change in time-use composition. This may be true especially for the participants having the measurements close to their retirement date as the timing of the measurements varied between the participants. In general, applying the same data as in this study we have previously observed short-term improvement in the domains of cognitive function during the transition to retirement (22). However, these immediate improvements are likely explained by other factors than changes in 24-h movement behaviors, e.g., removal of work-related stressors.

Our study has several implications. Among majority of retirees, sleep duration increases after transition from work to retirement (11,23), which may lead to negative consequences for reaction time based on our current findings. Preventing decline in reaction time in long-term is important because it reflects older adults’ capacity to solve everyday problems (51), drive a car (52), and slower reaction time is associated with increased risk for falls and chronic conditions (53,54). Therefore, it may be beneficial to promote recent retirees’ active lifestyle by increasing physical activity and other cognitive stimulating activities, so that increasing sleep does not replace these activities.

The strengths of this study include a longitudinal study design and annual data collections which allowed us to study concurrent, albeit short-term, changes in movement behaviors and cognitive function during retirement transition. Furthermore, applying compositional data analysis methods allowed us to account for multicollinearity between the time-use behaviors and to estimate effects on time reallocations between 24-h movement behaviors on cognition.

There are also limitations that should be taken into account when interpreting the findings. The relatively small study sample increases the risk of type II errors (failure to identify significant effect that actually exists). Since the change in 24-h movement behaviors and cognitive function was observed during the same period, we cannot be certain whether change in movement behaviors or cognition occurred first. Previous research has found a bidirectional association with cognitive function and physical activity (55). Thus, it is also possible that the improvement observed in most dimensions of cognitive function, but not in reaction time, during the retirement transition (10) may have driven an increase in physical activity. This may be explained by removal of psychological job strain after retirement and the fact that executive function is needed for planning and initiating physical activity (55). Future studies with longer follow-up are needed to elucidate the potential bidirectional relationship between the composition of 24-h movement behaviors and cognitive function. A further limitation was that we used self-reported data on sleep duration rather than objective measures. Although self-reported estimates of sleep duration are commonly used in studies, they often include periods of wakefulness as self-reports reflect time in bed rather than actual sleep time which may affect the observed associations between sleep and cognitive function. The ilr coordinate setup naturally impacts the interpretation of the findings. We chose the specific ilr coordinate set up to enable an examination of how the relationships between active and passive behaviors, LPA versus MVPA, and sedentary behavior versus sleep are associated with changes in cognition. Finally, given that the current study population consisted of mainly female, non-manual workers and the participants were physically relatively active and working until statutory retirement age, the generalizability of the findings may be limited.

## CONCLUSIONS

Reallocating time from sleep to sedentary behavior, LPA and MVPA during 1-yr transition from work to retirement was associated with an improvement in reaction time. No associations between changes in 24-h movement behaviors and other cognitive function domains were observed. Given that cognitive function declines with advancing age, future studies are needed to elucidate how changes in 24-h movement behaviors affect domains of cognitive function and risk of dementia in the long term.
